# Smartphone Apps for Surveillance of Gestational Diabetes: Scoping Review

**DOI:** 10.2196/38910

**Published:** 2022-11-21

**Authors:** Suzanne Smyth, Eimear Curtin, Elizabeth Tully, Zara Molphy, Fionnuala Breathnach

**Affiliations:** 1 Royal College of Surgeons in Ireland Dublin Ireland; 2 Rotunda Hospital Dublin Ireland

**Keywords:** gestational diabetes, digital health, mHealth, telemedicine, diabetes, apps, smartphone, remote feedback

## Abstract

**Background:**

Developments and evolutions in the information and communication technology sector have provided a solid foundation for the emergence of mobile health (mHealth) in recent years. The cornerstone to management of gestational diabetes mellitus (GDM) is the self-management of glycemic indices, dietary intake, and lifestyle adaptations. Given this, it is readily adaptable to incorporation of remote monitoring strategies involving mHealth solutions.

**Objective:**

We sought to examine and assess the available smartphone apps which enable self-monitoring and remote surveillance of GDM with a particular emphasis on the generation of individualized patient feedback.

**Methods:**

Five databases were searched systematically for any studies evaluating mHealth-supported smartphone solutions for GDM management from study inception until January 2022. The studies were screened and assessed for eligibility of inclusion by 2 independent reviewers. Ultimately, 17 studies were included involving 1871 patients across 11 different countries. The PRISMA-ScR (Preferred Reporting Items for Systematic Reviews and Meta-Analyses Extension for Scoping Reviews) conceptual framework was adhered to for data extraction and categorization purposes.

**Results:**

All studies analyzed as part of this review facilitated direct uploading of data from the handheld glucometer to the downloaded patient-facing smartphone app. Glycemic data were captured by all studies and were reassuringly found to be either improved or noninferior to extant models of hospital-based care. Feedback was delivered in either an automated fashion through in-app communication from the health care team or facilitated through bidirectional communication with the app and hospital portal. Although resource utilization and cost-effective analyses were reported in some studies, the results were disparate and require more robust analysis. Where patient and staff satisfaction levels were evaluated, the response was overwhelmingly positive for mHealth smartphone–delivered care strategies. Emergency cesarean section rates were reduced; however, elective cesarean sections were comparatively increased among studies where the mode of delivery was assessed. Most reviewed studies did not identify any differences in maternal, perinatal, or neonatal health when app-based care was compared with usual in-person review.

**Conclusions:**

This comprehensive scoping review highlights the feasibility, reliability, and acceptability of app-assisted health care for the management of GDM. Although further exploration of the economic benefit is required prior to implementation in a real-world clinical setting, the prospect of smartphone-assisted health care for GDM is hugely promising

## Introduction

Positive exploitation of the exponential growth and development seen in the information and communication technology (ICT) sector in the last decade has provided novel solutions to operational challenges such as overcrowding and staff shortages within the health care arena. Telemedicine has emerged as one such advancement and has seen rapid diffusion for the management of chronic diseases in particular. Diabetes is one such condition that has proven to be readily adaptable to self-management and remote monitoring.

Mobile health (mHealth) is a facet of telemedicine focusing on the use of mobile phone technology to facilitate exchange of health information between the patient and the caregiver. Increasingly, apps downloaded by the patient-user to personal handheld smart devices, such as phones or tablets, are used as data-capturing tools, conduits to share and exchange health information, and repositories of disease-specific information and education [1]. There are an estimated 3.8 million smartphone users in Ireland, representing an increase of 16.8% since 2018. Smartphone penetration rates Europewide reflect this trend, with projected ownership rates of 87%, 92%, and 94% by 2025 in France, Germany, and the United Kingdom, respectively [2].

This review sought to evaluate the smartphone apps that have been developed for gestational diabetes mellitus (GDM) to promote patient-centered care through the surveillance of markers of glycemic control, such as blood glucose levels, diet, exercise, and weight management. mHealth promotes a precision medicine model of care by maintaining channels of communication between patients and their health care professional while focusing the onus of disease management on the patients themselves. Such responsibility has been shown to foster improved patient compliance and satisfaction levels and represents an exciting new chapter in GDM care [3]. Demonstration of improved glycemic control has been shown from use of smartphone app–based interventions for adults with type 2 diabetes mellitus [4]. Treatment strategies for GDM and type 2 diabetes mellitus are similar, encompassing medical nutrition therapy, lifestyle modifications, and self-assessment of daily blood glucose levels, such that the patient with GDM is perfectly poised to benefit from app-assisted care.

Previous reviews have examined mHealth in the context of all types of diabetes rather than a specific focus on GDM [5]. Leblalta et al [6] addressed all digital health interventions available to support women with GDM, including models of web-based care that have arguably become outdated. We sought to refine this existing knowledge by exploring further and assessing the surveillance strategies and capabilities afforded by smartphone apps for women with GDM.

## Methods

### Search Strategy

A comprehensive literature search was conducted following consultation with a reference librarian. With the aim of evaluating smartphone apps used for the surveillance of GDM, we reviewed the following medical databases: PubMed, Embase, CINAHL, Web of Science, and the Cochrane library. All peer-reviewed literature in the English language and published between January 1990 and January 2022 was searched. Prior to 1990, the mobile phone was far more primitive and not capable of the technological features this review aimed to assess. The incorporation of ICT into health care management represented an area of rapid development during this time, with telemedicine emerging as a potentially feasible pathway for management of chronic diseases in 1990 [7]. Inclusion and exclusion criteria for this scoping review are presented in Table 1 using the population-concept-context framework recommended by the Joanna Briggs Institute (JBI) methodology for scoping reviews. We also considered the PICO (patient/population, intervention, comparison, and outcomes). framework for systematic reviews in establishing our research question (Table 2). Medical subject headings were used where possible in our database searches. “Gestational Diabetes” was used in combination with each of the search terms outlined in Multimedia Appendix 1. The JBI reviewers manual was adhered to in the development of our scoping review protocol, which is available on request from the corresponding author (SS).

**Table 1:** Inclusion and exclusion criteria following the population-concept-context criteria recommended by the Joanna Briggs Institute.

	Inclusion criteria	Exclusion criteria
Population	GDM^a^	Type 1 diabetes mellitus, type 2 diabetes mellitus
Concept	Smartphone apps associated with a hospital-based clinical portal facilitating remote monitoring	Smartphone apps for personal surveillance of GDM but with no oversight from the obstetric diabetes team through a hospital clinical portal
Context	Surveillance of at least 1 element of GDM care (glycemic indices, diet, exercise, weight, blood pressure)	Surveillance of additional parameters not primarily involved in GDM care (eg, psychological assessment, remote management of a separate obstetric condition)

^a^GDM: gestational diabetes mellitus.

**Table 2:** Characteristics of studies evaluating smartphone app–assisted care for GDM and the provision of remote feedback.

Article information	Type of trial	Participants, n	GDM^a^ diagnosis	Glycemic targets	Self-monitoring schedule	Details of smartphone app–assisted technology
Calle-Pascual et al, Spain (2010) [8]	Prospective randomized interventional study	100	Carpenter Couston criteria; <28 weeks’ gestation	Fasting <95 mg/dl; 1-h postprandial <120 mg/dl	6× a day	Infrared enabled transfer of SMBG^b^ from glucometer to smartphone app (preinstalled), with captured data then transferred to a central hospital database (Emminens Conecta Plus Web Application); bidirectional communication between patient and HCP^c^
MacKillop et al, UK (2018) [9]	Randomized controlled trial	206	Fasting >5.6 mmol/l; postprandial >7.8 mmol/l <35 weeks’ gestation	Fasting <5.3 mmol/l; 1-h postprandial; 7.8 mmol/l; 2-h postprandial <6.4 mmol/l	6× a day, 3 days a week	Bluetooth-enabled transfer of SMBG from glucometer to smartphone app (GDm-Health), with captured data transferred to a secure website. Review of website 3 × a week by the specialist midwife; unidirectional communication of staff to patient only
Guo et al, China (2018) [10]	Randomized interventional study	124	IADPSG^d^ criteria; 24-28 weeks’ gestation	Unspecified	6× a day, 3 days a week reducing to 2 days a week if control demonstrated	Automatic data upload from glucometer to app (Dnurse); HCP review of uploaded data from app to doctor-facing version of Dnurse; HCP able to communicate with patient to adapt medical guidance; unidirectional communication of staff to patient only
Al-ofi et al, Saudi Arabia (2019) [11]	Randomized open-label control study	60	IADPSG criteria; 24-28 weeks’ gestation	Fasting <5.1 mmol/l; postprandial <8.5 mmol/l	4× a day	Glucometer linked to smartphone app (Glucomail) enabling easy transfer of data to the app, with captured data then transferred to a secure hospital-based system; an immediate alert is generated to the HCP if above-threshold levels are recorded, allowing for further action to be taken; unidirectional communication of staff to patient only
Yew at al, Singapore (2021) [12]	Randomized controlled trial	340	WHO^e^ 2013 criteria (endorsed IADPSG criteria); 12-30 weeks’ gestation	Fasting <5.5 mmol/l; 2-h postprandial <6.6 mmol/l	7× a day, 2-3 times a week	Smartphone-based lifestyle coaching program associated with a secure web app (Habits-GDM); app-compatible glucometer to transfer SMBG values; bidirectional communication between patients and HCP
Borgen et al, Norway (2019) [13]	Randomized controlled trial	238	2-h OGTT^f^ >9 mmol/l; <33 weeks’ gestation	Unspecified	Unspecified	Bluetooth-enabled transfer of SMBG values from glucometer to app (Pregnant+); automated color-coded feedback in direct response to glycemic control; no in-app communication between patient and HCP
Sung et al, South Korea (2019) [14]	Randomized controlled trial	21	2-step approach IADPSG criteria or Carpenter Couston criteria; <30 weeks’ gestation	Unspecified	4× a day	Bluetooth-enabled transfer of SMBG values from glucometer to smartphone app; automatic transfer of data by a wireless network captured in the app to a secure server; bidirectional communication between HCP and patient; HCP sends tailored medical and nutritional guidance from the server to the app
Miremberg et al, United States (2018) [15]	Randomized controlled trial	120	2-step process Carpenter Couston criteria; <34 weeks’ gestation	Fasting <95 g/dL; 1-h postprandial <140 g/dL	4× a day	Delivery of personalized feedback from the HCP secure database to the patient’s app (Glucose Buddy) regarding self-management, glycemic control, and follow-up scheduling; bidirectional communication between the HCP and the patient.
Poulter et al, Australia (2021) [16]	Intervention study	100	IADPSG criteria; 24-30 weeks’ gestation	Fasting <5 mmol/l; postprandial <6.7 mmol/l	4× a day	Bluetooth-enabled glucometer to upload SMBG data to the app (NET Health) which are automatically sent to a secure central server, with the software server automatically flagging the above-threshold glycemic values; unidirectional communication of the HCP to patients through the in-app interface
Rigla et al, Spain (2018) [17]	Pilot study	20	NDDG^g^ criteria; <34 weeks’ gestation	Unspecified	4× a day	Bluetooth-enabled glucometer facilitating transfer of SMBG to the app (MobiGuide) with subsequent transfer of the data to a specifically designed decision support software, with the HCP using a web-based app to visualize all the patient data; no feedback between staff and patients through the app/server system
Varnfield et al, Australia (2021) [18]	Feasibility study	40	IADPSG criteria; 24-28 weeks’ gestation; (if RF^h^ at earlier OGTT in T1, with repeat at 24-28 weeks if normal)	Fasting <5 mmol/l; - h postprandial <7.4 mmol/l; 2-h postprandial <6.7 mmol/l	4× a day	Bluetooth enabled glucometer facilitating transfer of SMBG values to the app (MoTHER); automatic transmission of app data to the clinician web portal which is reviewed weekly by the HCP; no in-app communication between HCP and patients
Khalil et al, France (2019) [19]	Qualitative study	15	Unspecified	Unspecified	6× a day reducing to 3 × a day if stable BGL^i^	Bluetooth-enabled glucometer to facilitate transfer of SMBG to the app (MyDiabby); color-coded (green, orange, red) automated feedback reflecting glycemic control and customized alert system at the server/HCP end of the solution; bidirectional communication between HCP and patients
Moazen et al, Austria (2021) [20]	Pilot study	27	Unspecified	Unspecified	4× a day	Bluetooth-enabled glucometer to facilitate transfer of SMBG to the app (DiabCare); data are transferred from the app to an online data management system accessible by the health care team; bidirectional communication between HCP and patients.
Seo et al, South Korea (2020) [21]	Case series study	4	Diagnosed following OGTT at 24-28 weeks’ gestation	Unspecified	Unspecified	Bluetooth-enabled glucometer to facilitate transfer of SMBG to the app; transfer of data captured by the app via wireless network to the study server; personalized and automated feedback; bidirectional communication between HCP and patients
Wickramasinghe et al, Australia (2019) [22]	Randomized crossover study	10	Diagnosed following OGTT at 26-28 weeks’ gestation	Unspecified	4× a day	Bluetooth-enabled glucometer to facilitate transfer of SMBG to the app (Diamond solution); data captured by the app are reviewed on a secure platform by the HCP who responds to the patient with recommendations; unidirectional communication of the HCP to patient through the in-app interface
Yang et al, China (2018) [23]	Pilot intervention study	157	WHO 2013 criteria (endorsed IADPSG criteria)	Fasting <5.3 mmol/l; 1-h postprandial <7.8 mmol/l; 2-h postprandial <6.7 mmol/l	10× a day	Smartphone app using a WeChat system to which blood glucose, blood pressure, and weight are uploaded; data are subsequently uploaded to a cloud platform and are evaluated by the HCP through the HCP’s own WeChat interface; unidirectional communication of HCP to patient through the WeChat system
Tian et al, China (2020) [24]	Randomized controlled trial	309	IADPSG criteria; <31 weeks’ gestation	Fasting <5.3 mmol/l; 1-h postprandial <7.8 mmol/l; 2-h postprandial <6.7 mmol/l	5× a day for 6 days in a 2-week block	Smartphone app using a WeChat system to which blood glucose, diet, exercise and weight are uploaded; unidirectional communication of HCP to patient only; peer-to-peer communication

^a^GDM: gestational diabetes mellitus.

^b^SMBG: self-monitored blood glucose

^c^HCP: health care provider.

^d^IADPSG: International Association of Diabetes in Pregnancy Study Group.

^e^WHO: World Health Organization.

^f^OGTT: oral glucose tolerance test.

^g^NDDG: National Diabetes Data Group.

^h^RF: risk factors.

^i^BGL: blood glucose level.

### Outcome Measures

The primary outcome of this review was assessment and achievement of glycemic control following adoption of app-assisted health care delivery focusing on personalized or automated feedback of at least 1 component of standard GDM surveillance. Secondary outcome measures included patient and staff satisfaction levels and the cost-effectiveness of app-based interventions.

### Screening

**Figure 1:**
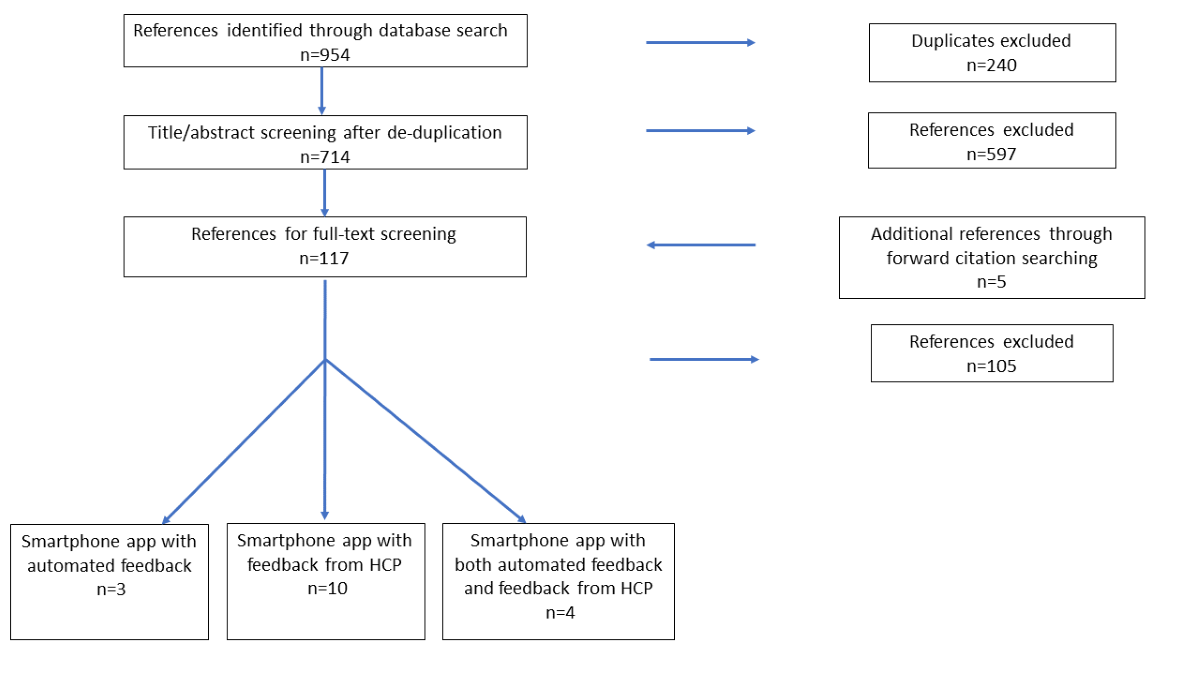
PRISMA flow diagram describing the systematic literature search for studies examining the effect of smartphone app-assisted care associated with remote feedback for GDM.

Guidelines from the PRISMA-ScR (Preferred Reporting Items for Systematic Reviews and Meta-Analyses Extension for Scoping Reviews) were adhered to during the literature search and screening process [25]. Retrieval of titles from database searching as described in the previous section was performed independently by 2 authors (SS and EC). These same 2 authors independently screened the titles and abstracts generated by the search to assess fulfilment of the study inclusion criteria. Relevant studies meeting the inclusion criteria were selected for review in this study. A third reviewer (FB) was available to oversee discussions pertaining to discrepancies which were solved by consensus opinion. The initial search strategy yielded 954 articles which were subsequently refined such that 15 articles were included in the final review. A schematic representation of the screening process is depicted in Figure 1.

### Data Extraction

A data extraction form was developed to collate and record information from each article that would later inform data synthesis. Similar to the screening process described above, the PRISMA-ScR conceptual framework was employed to achieve extraction and categorization of data while subsequently facilitating inferences and conclusions to be drawn from it. The data extraction form was designed to capture the following three criteria: (1) publication characteristics, including authorship, study title, year of publication, journal of publication, and country of origin; (2) characteristics of the app-assisted care program and details of any remote monitoring systems; and (3) study outcomes, including achievement of glycemic control, staff and patient-user satisfaction, and cost-effectiveness.

The data extraction tool was sampled on a random subset of 3 papers and was later refined to ensure all desired elements were captured.

### Synthesis of Results

Following data extraction, a validation check was completed, after which the data from each article were summarized and presented in narrative fashion. Key characteristics of the smartphone app–assisted interventions were recorded along with outcome data, which were subcategorized as follows: glycemic control; resource utilization and cost; satisfaction of key stakeholders; and maternal, perinatal, and neonatal outcomes.

The aim of this scoping review was to give a transparent, systematic overview of existing remote management strategies involving smartphone app–assisted care for GDM management. As such, neither methodological quality nor risk of bias was critically appraised as part of the review.

## Results

The electronic database search yielded 954 results. Subsequent removal of duplicates resulted in 714 articles for title and abstract screening. A further 597 references were then excluded, as they did not meet the inclusion criteria. The remaining 117 articles were subject to full-text screening. An additional 5 studies obtained following forward citation searching were also screened by full-text review. This process resulted in the exclusion of 105 articles. Excluded studies included those describing the use of mobile phone technology without specific use of a smartphone app, descriptions of software and technological architecture development, and studies relating solely to web-based interventions (Textbox 1). Ultimately, 17 articles were included in the final review.

**Reasons for study exclusion following full-text review.**

**Excluded studies following full-text review**

- Phone use but not use of a smartphone app (n=18)
- Description of software development (n=18)
- Web-based interventions only (n=19)
- Smartphone apps that functioned as a repository only (n=6)
- Poster abstract only (n=4)
- Not assessing gestational diabetes mellitus (n=3)
- Secondary analyses (n=26)
- Inaccessible (n=8)

There were 17 studies included in this review, the characteristics of which are represented in Table 2. The included studies were published between 2010 and 2022, with the majority (16/17, 94%) published during or since 2018. The studies were conducted across 11 countries, with 7 from Asia, 6 from Europe, 3 from Australia, and 1 from the United States. There were 10 randomized controlled trials (RCTs), 5 pilot intervention studies, 1 qualitative study, and 1 case series. The number of participants ranged from 4 participants in the case series to 340 in the largest of the RCTs (mean 111; median 100).

### Description of the Smartphone-Assisted Remote Monitoring Solutions for Surveillance of GDM

All included studies reported direct uploading of data from the glucometer to the smartphone app. Although one early study used infrared transfer of data, Bluetooth-enabled transfer of glycemic indices was the most common approach. All studies involved the use of a smartphone app. Study participants were provided with a smartphone on which the study app was preinstalled in 2 studies, but in all other cases, the patient’s personal smartphone was used. Pervasive management solutions compatible with several operating systems, such as Android and iOS, were used in 13 studies [10-16,18-20,22-24].

The most commonly captured variable in the smartphone apps was glycemic data, which all 17 described app-assisted care programs had the capability of performing. Other tracked variables included dietary and lifestyle information, weight, medication dosing, blood pressure, ketonuria, and heart rate.

Automatic transfer of data captured in the app was sent to a secure hospital-based server through a wireless network in 14 of the reviewed studies. Of the remainder, 2 studies used the WeChat app to allow cloud storage of patient data only accessible to the research team [23,24]. One app-assisted care pathway was not linked with a hospital-based server and thus did not have real-time remote monitoring capabilities [13].

### Description of Personalized Health Care Provider–Delivered Feedback

Bidirectional communication of data, questions, and advice was available between patients and their obstetric diabetes teams in 8 of the reviewed smartphone app–linked telemedicine systems [8,12,14,15,19-21,24]. A further 6 of the reviewed studies demonstrated the capability of in-app communication delivered from a member of the health care delivery team to the patient [9-11,16,22,23]. The 3 remaining studies provided automated feedback to the patient [13,17,18].

### Description of Automated Feedback and Messaging

The generation of automatic feedback by the server to the app in specific response to uploaded patient data was noted in 4 studies [10,13,17,19]. In 2 studies, this feedback was represented pictorially in a color-coded traffic light system, with green icons signifying a normal result and red icons signifying an above-threshold glucose result [13,19]. A patient-facing alert in the form of a pop-up message was generated in the setting of above-threshold readings in the other 2 studies [10,17]. Patients were prompted and directed toward a questionnaire link for completion to elaborate on potential causative lifestyle factors.

A further 4 studies issued in-app educational information and motivational pop-up messages. Although these were not specifically tailored to a woman’s uploaded app data, they served to reinforce the monitoring strategies for GDM and highlight the importance of achievement of appropriate glycemic control [11,14,18,21]. One study reported the generation of in-app prompts reminding participants to capture a 7-point capillary glucose profile on any 2 days of the week [12].

### Outcomes

#### Glycemic Indices

Glycemic indices were the most commonly reported upon clinical outcome data. Improved management of glycemic indices by app users was demonstrated in 9 studies [10-12,15,20-24], while noninferior glycemic control, manifested by self-monitored finger prick blood indices or hemoglobin A1C, was noted in a further 2 studies [8,9]. Moreover, 2 studies assessed app-assisted care based on postnatal assessment of glycemic control [13,14]. Although lower rates of insulin resistance were demonstrated by one of these studies, this did not reach statistical significance. The second study assessing a 2-hour postnatal oral glucose tolerance test did not report any significant difference when compared with the control group.

#### Resource Utilization and Cost Analysis

Resource utilization was reported in 4 studies, the majority of which (n=3) reported a reduction in unscheduled hospital attendances by app-using participants [8,10,16]. One study reported the converse, with an increased number of low-utility clinic visits when an app-assisted pathway of care for GDM was compared with a historical control [18]. The authors surmised that this might be explained by an increased level of self-monitoring prompting patients with above-threshold readings to present for review. Cost-effectiveness of smartphone app–assisted care delivery was considered in 2 studies [9]. No significant cost saving was demonstrated in the economic analysis of one study, whereas the other study reported a cost saving of Aus $23 (US $15.32) per patient reflected by 37 minutes of total clinician time saved in the app-using group compared with the control group.

#### Satisfaction With Smartphone App–Assisted Care

Patient satisfaction was explored in 6 of the studies [9,16-19,22]. A further 2 studies reported increased compliance levels with self-monitoring schedules, and satisfaction could be inferred from such usage behavior [10,15]. Staff satisfaction with this remote model of care provision was evaluated in 3 studies, and all were overwhelmingly in favor of the transition [18,19,22].

#### Maternal, Perinatal, and Neonatal Outcomes

Data pertaining to maternal, perinatal and neonatal outcomes were reported in 11 of the 17 (65%) reviewed studies [8-13,15-17,23,26]. One RCT found that women in the app-assisted care delivery group had fewer cesarean sections than did the comparator group (*P*=.005) [9]. In another study which reported no difference in mode of delivery between an app-using group and a historical control cohort, the authors did note fewer emergencies but a greater number of elective cesarean sections among the app-using women [18]. An increase in elective cesarean sections was similarly noted in another study, and this was associated with a *P* value of <.05 [23]. Two studies reported reduced weight gain while another study reported reduced blood pressure in their respective intervention groups [10,11,17]. No differences in maternal or perinatal outcomes were demonstrated across the other studies. The results of the majority of studies looking at neonatal outcomes were noninferior for app use compared with standard care, but one study did demonstrate fewer composite adverse neonatal outcomes among app-using participants (*P*=.006) [12].

## Discussion

This scoping review provides a comprehensive overview of the availability and functionality of smartphone apps capable of the generating remote feedback in the surveillance of women with GDM. We have noted that app-assisted care is noninferior to standard clinic-based care in terms of glycemic treatment targets, and in fact, half the reviewed studies identified an improvement in overall glycemic control [10-12,15,20-24]. Such evidence demonstrates the feasibility of adopting app-assisted health care for GDM.

If the adoption and diffusion of app-assisted platforms as a viable aspect of surveillance are to be successful, patient and staff satisfaction and acceptability levels must be high. Continued use of novel solutions in health care management, such as smartphone apps, requires accessible, easily interpretable, and aesthetically pleasing interfaces. Additionally, behavioral intention has been highlighted as a significant determinant of ongoing health technology use and engagement by the patient [27]. Other factors that should be taken into consideration in the development and dissemination stage of artificial intelligence–assisted technologies are personal innovativeness or the willingness to engage in a new health solution as well as performance and effort expectancy [27-29]. In this review, we have shown that over half of the reviewed studies (10/17, 58 %) did not seek to assess patient or staff satisfaction levels, although we acknowledge that secondary analyses might have explored these themes. Three studies did assess patient compliance with the mandated monitoring schedule and thus satisfaction can be inferred, although not proven, from continued usage behavior in these studies [10,15,24]. Where satisfaction was assessed, all studies reported positive experiential expressions from the app-using groups [9,16-19,22]. Such expressions included reassurance that blood glucose levels were being reviewed frequently, and often in real time, by the obstetric diabetes team and feelings of self-efficacy, autonomy, and convenience. Satisfaction among staff users of the app-linked technologies was only assessed in 3 studies. Effective and time-efficient management was the most commonly identified theme. Ensuring collaboration and endorsement between all stakeholders in a novel ICT-based health intervention is crucial to its success. Cognitive trust has been found to be an impacting factor on the behavioral intentions of physicians’ use and endorsement of app-assisted health care [30]. Robust RCTs prior to mass product circulation will contribute to allaying trust concerns with the technology.

The impact of resource utilization and economic benefit have been promoted as hugely beneficial effects of telemedicine, and by extension, so have mHealth management strategies [31,32]. These themes were only explored in 5 of the studies [8-10,16,18]. A statistically significant reduction in resource use by app-using women was noted by one study, and this reduction resulted in an overall cost saving for the hospital [16]. An analysis by another study, however, did not report significant cost savings [9]. Finally, one study reported an increase in the number of low-utility clinic visits among app users compared with a historical control group [18]. This may be a result of increased compliance with self-monitoring leading to a greater numbers of hyperglycemic episodes that need to be evaluated. This particular study did not offer feedback relating to uploaded glycemic indices or lifestyle patterns, and addition of these capabilities to the app technology would likely have an impact on requirement for in-person hospital review. Such considerations should be given due attention during early iterations of the app development phase.

The evidence collated in this review demonstrates achievement of equivalent or improved glycemic control, confirms noninferior maternal and neonatal outcomes, and highlights the potential for reduced resource utilization and economic efficiency among women availing of app-assisted health care delivery for GDM. The transition towards incorporation of mHealth technologies such as smartphone apps has been welcomed by women with GDM who have shown high levels of satisfaction with a self-monitored remote management strategy. Key to the success of such smartphone apps is the maintenance of communication between the patient and her obstetric diabetes team. To optimize this alliance, a bidirectional communication strategy, as described in 8 of the studies in this review, would likely help to obviate the requirement for many women with well-controlled GDM to attend the hospital for in-person review without impacting on their sense of team involvement or hospital oversight. The resource implications of such a strategy are not limited to the hospital infrastructure alone, with benefits envisaged for the patient through the potential avoidance of financial and time constraints associated with frequent hospital attendances.

Given the rapid expansion of the telehealth sector, this review of app-assisted health care facilitating remote feedback in the setting of GDM is timely and judicious. However, to allow for comprehensive knowledge acquisition of the potential benefits and drawbacks of telemedicine and mHealth management of GDM, further research is still required. For instance, only 1 study in this review adapted a smartphone app for a culturally diverse audience [13]. In an era of great ethnic diversity within populations, absence of such a feature could be an exclusory factor. Further, patients who are willing to partake in research studies are more likely to be health and eHealth literate which may introduce bias into the study cohorts. Finally, the impact of app-assisted health care and remote surveillance needs robust health economic assessments to enable the refinement of existing technology such that app-assisted systems can viably become integrated into routine medical practice.

## Appendix

Multimedia Appendix 1 Search terms.

## Abbreviations

GDM: gestational diabetes mellitus
ICT: information and communication technology
JBI: Joanna Briggs Institute
mHealth: mobile health
PICO: patient/population, intervention, comparison, and outcomes
PRISMA-ScR: Preferred Reporting Items for Systematic Reviews and Meta-Analyses Extension for Scoping Reviews
RCT: randomized controlled trial
