# Supplementary material for: Smartphone Apps for Surveillance of Gestational Diabetes: Scoping Review
Source: JMIR Diabetes. 2022 Nov 21;7(4):e38910. doi: 10.2196/38910 (PMC9723973; doi:10.2196/38910)
Supplement: Multimedia Appendix 1 [file diabetes_v7i4e38910_app1.docx]

Appendix 1 Search Terms:

App

Smartphone application

M-health

E-health

Telehealth

Telemedicine

Telemonitoring

Internet

Computer

Bluetooth

Phone

Mobile Phone

Digital health

Media

Social media

SMS

Text message

Messaging

Wireless

Remote-monitoring

Self-management

Remote surveillance

Machine learning

Wearable devices

Big data

AI

Artificial intelligence
